# Sex-specific characteristics of special endurance and performance potential in female runners

**DOI:** 10.1186/s13104-025-07256-6

**Published:** 2025-04-26

**Authors:** Wolfgang Blödorn, Frank Döring

**Affiliations:** 1 Lindenstraße 2, 24211 Preetz, Germany; 2 Institute of Human Nutrition and Food Science, Department of Molecular Prevention, Christian-Albrechts-University of Kiel, Heinrich Hecht-Platz 10, 24118 Kiel, Germany

**Keywords:** Special endurance, Running performance, Female runners, Track running, Sex-specific differences

## Abstract

**Objective:**

The coefficient of special endurance (KsA) is a metric that quantifies the relative pace loss between two consecutive distances (e.g., 100 m/200m). Here, we analyzed over 20,000 race times to determine KsA values for female runners across seven distance pairs from 100 m to 10,000 m. The data analyses are based on multiple official performance rankings at international to regional levels, exclusively compiled and processed for this study.

**Results:**

The KsA values obtained have remained stable for over four decades in national-level female runners and are applicable from world-class to regional levels. A sex-based analysis reveals that females undergo a more pronounced decrease in pace from 100 m to 1500 m in comparison to males. These sex differences in special endurance align with known variations in muscle fiber composition and fast-twitch type II fiber characteristics between males and females. In conclusion, we provide statistically valid KsA reference values for female runners from 100 m to 10,000 m. These values have practical implications for coaches and athletes seeking to assess runners’ strengths, weaknesses, potential, and specific talents based on race times. Sex differences in KsA values may reflect muscle physiology and guide future research on KsA and muscle function.

**Supplementary Information:**

The online version contains supplementary material available at 10.1186/s13104-025-07256-6.

## Introduction

An analysis of running performance highlighted a negative exponential relationship between pace and distance, consistent with world records for both sexes [1]. Physiological and theoretical models [2, 3] based on bioenergetics (e.g., aerobic capacity) partially explain and predict running performance across different performance levels in both males and females [1]. Recently, we proposed a statistical approach to analyze the running performance of males based on Coefficients of Special Endurance (KsA) [4, 5]. These easy-to-use values serve as metrics that quantify the relative pace loss between seven neighboring race distances (100 m/200m to 5000 m/10,000 m). Here, we conducted a similar analysis for female runners to examine their characteristic pace loss, to assess individual performance potential, and to gain insights into sex differences in special endurance.

## Materials and methods

The sources of datasets Table S01 to Table S15 are as follows: The German Athletics Association (DLV) outdoor athletics rankings for female athletes from 1980 to 2022 were used to compile datasets S01–S07. For the years 1980 to 2009, we used the printed versions of the annual best lists, while for the years 2010 to 2022, we used the electronic versions available at www.leichtathletik.de. The open-source performance statistics website of World Athletics (www.worldathletics.org) was used to compile the first 300 female all-time outdoor top lists from 100 m to 10,000 m at the global (S08), European (S09), and British (S10) levels. The female all-time outdoor top lists for the same distances were also retrieved for Germany (S11; including West and East Germany) and for two German states, Baden-Württemberg (S12) and Schleswig-Holstein (S13), from the following websites: www.leichtathletik-dgld.de, www.blv-online.de, and www.shlv.de. Datasets S14 and S15 were compiled based on datasets S08–S13.

The datasets for female runners were collected similarly to those for male runners [4, 5]. The annual outdoor top-30 lists of the German Athletics Association (DLV) were screened for female runners listed in neighboring distances (100 m/200m to 5000 m/10,000 m) from 1980 to 2022, yielding 3260 distance pairs from 10,080 performances (Tab. S01–S07). Female runners’ personal bests, listed over neighboring distances, were taken from the all-time rankings (first 92 to 300) for the World (until 07/2023), Europe (07/2023), Great Britain (07/2023), Germany (West/East; 12/2022), and two German states (Baden-Württemberg 12/2021, Schleswig-Holstein 06/2023) (Tabs. S08–S13). After removing duplicates, 10,423 performances yielded 3427 distance pairs (Tabs. S14). Female middle-distance runners, categorized by absence or presence in the 3000 m rankings, are shown in Table S15.

**Fig. 1:**
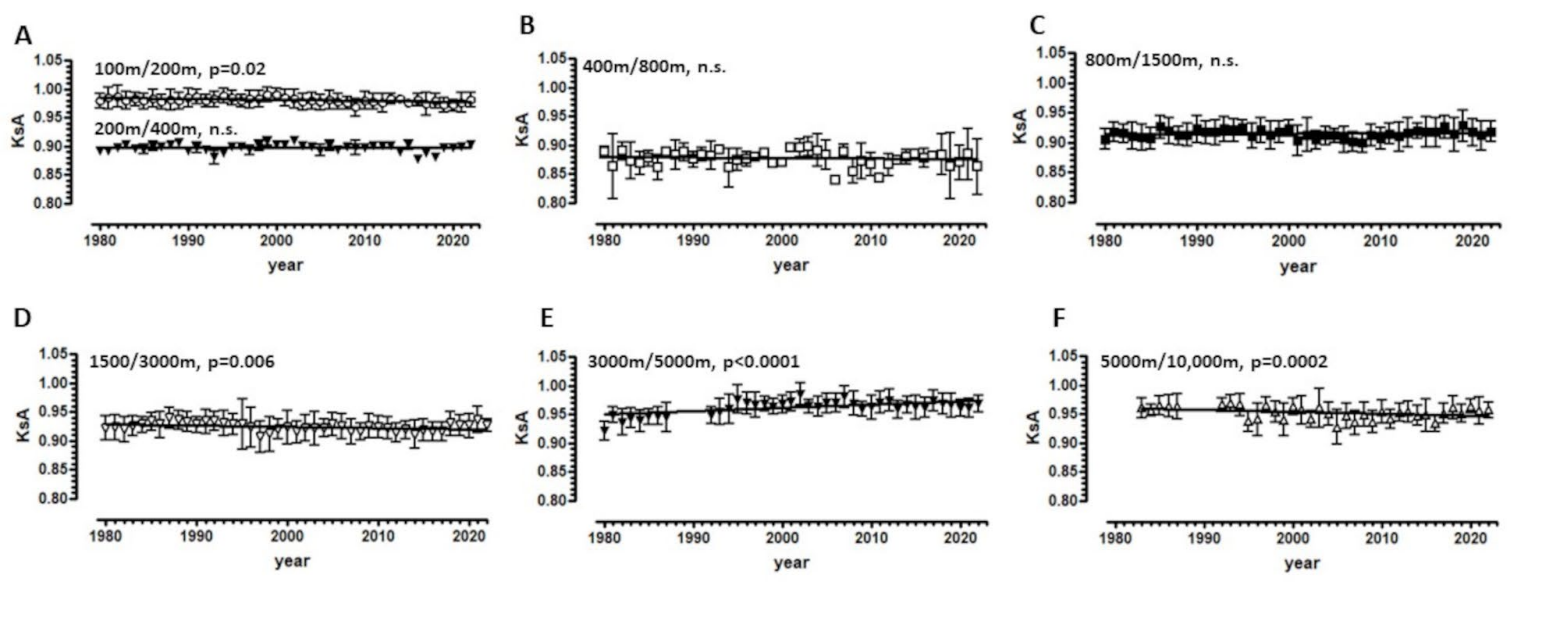
Changes in KsA values (mean ± SD) for pairs of neighboring distances (**A**-**F**) between 1980 and 2022, based on annual best performance times of German female runners. *p*-values indicate the significance of the slope deviation from zero. n.s., not significant. Datasets: Tab. S01-S07

**Fig. 2:**
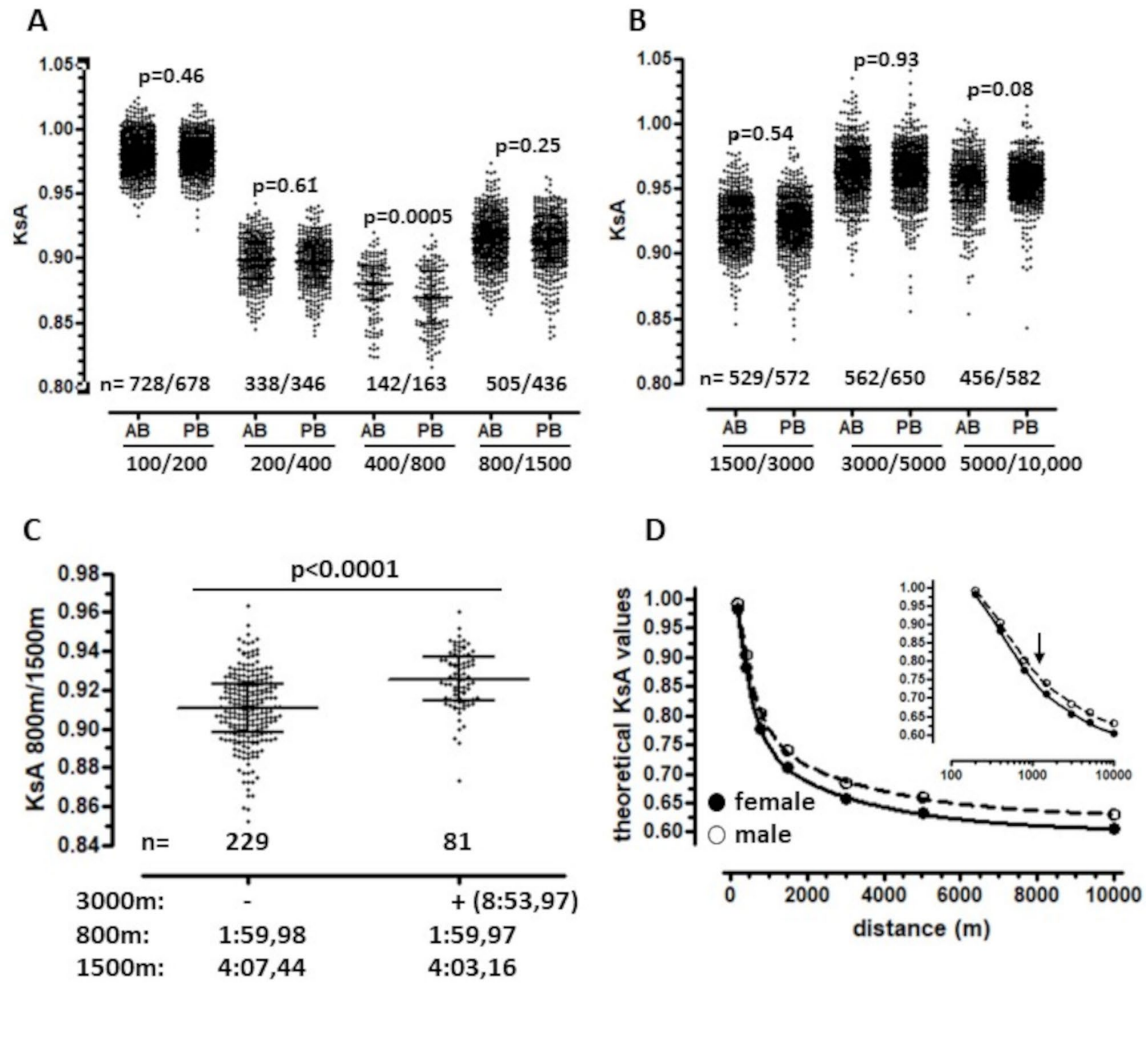
Distribution of KsA values for neighboring distances (in meters; A/B), KsA values for middle-distance runners (**C**), and the trajectory of theoretical KsA values as a function of distance (**D**). (**A**, **B**) Data points represent KsA values (median, interquartile range) derived from annual best times of German female runners (AB) or personal best times of female runners from regional to world-class level (PB). *n* denotes the number of KsA values. *p-values*: Mann-Whitney U test. Datasets: Tab. S01–S14. (**C**) Data points represent KsA values (median, interquartile range) derived from personal bests of female middle-distance runners, categorized by absence (-) or presence (+) in the 3,000 m rankings. *n* denotes the number of KsA values. p-value: Mann-Whitney U test. Dataset: Tab. S15. (**D**) The two-phase exponential function was derived by successive multiplying median KsA values (Table 1, annual best times of German female runners). Inset: Same data with log-transformed distance. Arrow indicates a breakpoint in curve trajectory. Respective data for male runners are shown [5]

Statistical methods followed those used for male runners [5]. KsA calculations and non-statistical analyses were performed in Excel, while statistical tests and graphs were generated in GraphPad Prism. A p-value of less than 0.05 was considered significant. Descriptive statistics (Table 1) were calculated using standard formulae. Linear regression was utilised to assess trends in KsA values over time (Fig. 1), with the objective of testing whether the slope differed from zero. The normality of distributions of KsA values (Fig. 2A-C) was tested using the Shapiro-Wilk test, and where significant deviations were noted, the Mann-Whitney U test was used. Theoretical trajectory of KsA values were obtained by multiplying median KsA values (Table 1) resulting in a two-phase exponential decay function (Fig. 2D).

**Table 1:** Descriptive statistics of KsA values for pairs of neighboring distances, derived from annual best times of German female runners between 1980 and 2022

distance (m) pair n		100/200	200/400	400/800	800/1500	1500/3000	3000/5000	5000/10,000
		728	338	142	505	529	562	456
**KsA**	**mean**	0.981053	0.897858	0.877443	0.914277	0.924515	0.963167	0.953554
	**SD**	0.014541	0.018650	0.021839	0.020485	0.021362	0.021303	0.021902
	**CV (%)**	1.48	2.08	2.49	2.24	2.31	2.21	2.30
	**95% LCL**	0.979995	0.895863	0.873820	0.912486	0.922691	0.961402	0.951539
	**95% UCI**	0.982111	0.899854	0.881066	0.916068	0.926340	0.964932	0.955570
	**Minimum**	0.933113	0.844453	0.822814	0.856831	0.845397	0.883901	0.853355
	**P25**	0.971858	0.884594	0.866751	0.900345	0.909699	0.950438	0.940501
	**median**	0.981388	0.898351	0.880397	0.914834	0.925713	0.963336	0.955544
	**P75**	0.989856	0.912507	0.893546	0.927727	0.939135	0.976573	0.967462
	**P90**	0.999748	0.920730	0.903088	0.939663	0.950777	0.988415	0.979652
	**Maximum**	1.024741	0.942662	0.919583	0.974134	0.990270	1.035461	1.021598
	^**1**^ **males**	0.992446	0.912000	0.887940	0.921097	0.923294	0.967013	0.953731

SD, standard deviation; CV, Coefficient of variation; LCL, lower confidence limit; UCL, upper confidence limit; P25/75/90, 25th /75th /90th percentile; [1]For comparison, the respective median KsA values for males are shown; [5] underlying datasets: Table S01-S07. The sources of the datasets are provided in the Materials and Methods section

## Results

For a given neighboring distance pair (e.g., 100 m/200m), the Coefficient of Special Endurance (KsA) is defined as the ratio of the pace at the shorter distance to the pace at the longer distance, thereby quantifying relative pace loss [4, 5]. The present study analyzed KsA values for seven distance pairs (100 m/200m, 200 m/400m, 400 m/800m, 800 m/1500m, 1500 m/3000m, and 5000 m/10,000 m) based on the annual best times of German female runners from 1980 to 2022. The analysis revealed that KsA values for the 200 m/400m and 400 m/800m distances did not exhibit a discernible annual trend (Fig. 1; sources of the data: Tabs. S01-S07). The remaining pairs demonstrated statistically significant trends. These fluctuations were minimal, with changes less than 0.1% and low correlation coefficients (r² < 0.1). Consequently, it can be concluded that the KsA values of German female runners have remained remarkably stable over a period exceeding four decades.

The annual KsA values for the seven distance pairs were aggregated, resulting in inter- and intra-individual KsA values exhibiting small interquartile ranges (< 3%), low coefficients of variation (< 2.5%), narrow 95% confidence intervals, and low standard deviations across all distance pairs (Table 1; sources of the data: Tabs. S01-S07). These KsA values do not differ significantly from those derived from the personal bests of female runners from world-class to regional level, except for the 400 m/800m pair (Fig. 2A/B; sources of the data: Tab. S01-S14) The utility of KsA values is further substantiated by the observation that female middle-distance runners who also ranked in the 3000 m exhibited significantly higher KsA values for the 800 m/1500m pair and are more than 4 s faster over 1500 m than those not listed in the 3000 m rankings (Fig. 2C).

The analysis of sex differences in KsA values indicate that females experience greater pace loss than males (Table 1, last line; sources of the data: S01-S07) in the 100 m/200m (1.11% points), 200 m/400m (1.36), 400 m/800m (0.75), and 800 m/1500m (0.63) pairs. The KsA values for 1500 m/3000m and 5000 m/10,000 m distance pairs were found to be similar between the sexes. Utilising the median KsA values of females (this study) and males [5], two-phase exponential decay functions were derived to model sex differences in pace loss across the entire distance range from 100 m to 10,000 m (Fig. 2D). The log-transformed functions support world record analyses (9), demonstrating two scaling laws for pace loss with a ~ 1000 m breakpoint. (Fig. 2D, inset). Overall, female runners experience a greater loss of pace than male runners for all distance pairs up to 1500 m.

## Discussion

Like male KsA values [4, 5], the respective female values have also remained stable for over four decades with minimal variation. Existing approaches (e.g., power law, physiological, and theoretical models) capture general pace loss patterns but may overlook sex-specific differences [2, 3, 6]. Our approach meticulously analyses pace loss from 100 m to 10,000 m in both males and females and should also be applicable to juniors and road runners. This facilitates accurate individual assessment of runners in a sex-specific manner. Our findings indicate that female runners experience a greater pace loss compared to their male counterparts from 100 m to 1500 m. In accordance with this, sex differences in world record times increase from sprints to middle distances, while remaining constant at longer distances [7, 8]. 

Sex differences in pace loss from 100 m to 1500 m are likely to have a physiological basis. Compared to females, males have larger lungs and hearts, higher oxygen transport capacity, greater muscle mass, particularly in the upper body, and more powerful type II fibers [9, 10]. In addition, their relative anaerobic energy supply is generally higher [11]. These factors are hypothesised to contribute to males´ superior anaerobic performance (100–800 m/1500m) and may provide a rationale for the observed lower pace loss (higher KsA values) up to 1,500 m. In contrast, females are hypothesised to benefit from a greater proportion of type I fibres and lower body weight, providing an endurance advantage. These sex-specific characteristics may balance out, resulting in similar KsA values between males and females beyond the 1500 m distance. Overall, sex-specific KsA values likely reflect underlying differences in muscle physiology between males and females, guiding future research on the relationship between KsA values and muscle function.

We provide statistically valid KsA values for female runners, quantifying pace loss between consecutive distances. As already shown for male runners [4, 5], KsA values are valuable for coaches and athletes. Since KsA values form a continuous chain of pace reductions from 100 m to 10,000 m, a runner’s main competition distance (e.g., 1500 m) can be evaluated in relation to performances at shorter (400 m, 800 m) and longer (3000 m, 5000 m) neighboring distances. Performance at shorter distances is particularly crucial, as it sets the upper limit for potential performance in the main event, while longer distances either enhance or diminish this influence. Thus, KsA reference values provide a simple, race-time-based tool for daily use in running practice, helping coaches and athletes to assess runner´s strengths, weaknesses, potential, and specific talents.

### Limitations of the study

One limitation of our study is the relatively small number (3.4 on average for each year from 1980 to 2022; 142/163 for annual and personal best times, respectively) of female performances for the 400 m/800m distance pair (see Fig. 1B/2A). This makes the 400 m/800m KsA value less certain than those for other distance pairs. The number of female runners ranked in both the 400 m and 800 m is generally limited worldwide, especially when considering 400 m performances of 53 s or faster. Another limitation concerns the KsA values for the 5000 m/10,000 m pair. The number of 10,000 m races is relatively low, suggesting that performance times over this distance may not yet be fully optimized. While this assumption is plausible, the similarity of KsA values between world-class and regional-level female runners (see Fig. 2B) indicates that the KsA values for 5000 m/10,000 m are statistically robust. Overall, future studies should pay more attention to the KsA values for the 400 m/800m distance pair. This is particularly important as the progression of 800 m times at the world-class level suggests that the 400 m times of elite female runners will increasingly shift toward 50–52 s.

## Conclusion

The present study provides KsA reference values for female runners quantifying pace loss from 100 m to 10,000 m and applying to all levels from world-class to regional. These values offer practical implications for coaches and athletes seeking to assess runners’ strengths, weaknesses, potential, and specific talents based on race times. Thus, the KsA approach is an easy-to-use tool for evaluating running performance in a sex-specific manner.

## Electronic supplementary material

Below is the link to the electronic supplementary material.

Supplementary Material 1

Supplementary Material 2

Supplementary Material 3

Supplementary Material 4

Supplementary Material 5

Supplementary Material 6

Supplementary Material 7

Supplementary Material 8

Supplementary Material 9

Supplementary Material 10

Supplementary Material 11

Supplementary Material 12

Supplementary Material 13

Supplementary Material 14

Supplementary Material 15

## Data Availability

All datasets used and analyzed in this study are publicly accessible and provided as supplementary tables to the manuscript.
